# Drugging Fuzzy Complexes in Transcription

**DOI:** 10.3389/fmolb.2021.795743

**Published:** 2021-12-21

**Authors:** Bonnie G. Su, Matthew J. Henley

**Affiliations:** ^1^ David H. Koch Institute for Integrative Cancer Research, Massachusetts Institute of Technology, Cambridge, MA, United States; ^2^ The Broad Institute of MIT and Harvard, Cambridge, MA, United States; ^3^ Department of Biology, Massachusetts Institute of Technology, Cambridge, MA, United States

**Keywords:** transcription factors, drug discovery, biophysics, chemical biology, fuzzy protein protein interactions

## Abstract

Transcription factors (TFs) are one of the most promising but underutilized classes of drug targets. The high degree of intrinsic disorder in both the structure and the interactions (i.e., “fuzziness”) of TFs is one of the most important challenges to be addressed in this context. Here, we discuss the impacts of fuzziness on transcription factor drug discovery, describing how disorder poses fundamental problems to the typical drug design, and screening approaches used for other classes of proteins such as receptors or enzymes. We then speculate on ways modern biophysical and chemical biology approaches could synergize to overcome many of these challenges by directly addressing the challenges imposed by TF disorder and fuzziness.

## Introduction

Gene regulation by DNA binding transcription factors (TFs) is fundamental to the identity, fate, and response to internal and external stimuli of eukaryotic cells (Lambert et al., 2018). By binding to sequence-specific sites on the genome and recruiting the transcriptional apparatus, TFs form the basis of selective gene control. Perhaps unsurprising given its central importance, gene dysregulation is a common driver across a wide variety of diseases and thus modulating transcription is a common objective in many drug discovery campaigns (Lee and Young, 2013; Henley and Koehler, 2021). However, the most direct and specific way to modulate transcription—by directly targeting the function of individual TFs that regulate disease-driving genes—is historically one of the most intractable endeavors in drug discovery.

In order to promptly respond to cellular stimuli, TFs must be capable of rapidly recruiting multiple distinct co-regulators to transient complexes, often using only a single short domain (Sigler, 1988). TFs accomplish this by forming an ensemble of malleable structures that can be adapted to different interfaces, facilitating many specific but highly disordered “fuzzy” interactions with binding partners (Brzovic et al., 2011; Fuxreiter, 2012; Tuttle et al., 2018; Teilum et al., 2021). This is a major contributor to flexible, context-dependent transcriptional regulation.

As a consequence of their disorder, TFs are largely considered an “undruggable” protein class (Darnell, 2002; Bushweller, 2019; Henley and Koehler, 2021). Fuzzy transcriptional PPIs have not yet been successfully targeted with drugs or even bona fide chemical probes; instead, virtually all the progress against TF PPIs in drug discovery has taken advantage of well-structured regulatory interactions that are specific to individual TFs or TF families (Henley and Koehler, 2021). To truly take advantage of the direct access to disease processes that TFs provide, it will be necessary to be able to target the TF functions that are dominated by disorder and fuzziness.

### Fuzziness and Druggability: Why Are TFs Difficult Targets?

**FIGURE 1:**
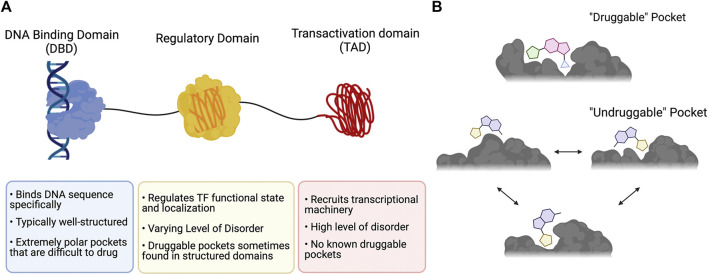
The druggability of transcription factors. **(A)** Typical domain organization of a TF annotated with key points about function and druggability. **(B)** Comparison of a prototypical “druggable” pocket that is deep and well-defined with an “undruggable” pocket that is characteristic of a fuzzy PPI interface and is dynamic, poorly defined, and shallow. This figure was created using Biorender.com.

One of the major challenges with targeting TFs is the absence of structured binding pockets. Targeted small molecule drug discovery requires pockets within a protein that can bind to small molecules and perturb the protein’s function. These pockets are typically deep, well-defined hydrophobic crevices that are directly involved with a protein’s function (Figure 1A) (Scott et al., 2016). Such “druggable” pockets are commonly found in enzymes and receptors, many of which naturally bind small molecules in their active sites. Conversely, most TFs lack these pockets, as their main functions are to form protein-protein and protein-DNA interactions. TF PPIs occur over large shallow surfaces, and the most critical PPIs (e.g., engagement of the transcriptional apparatus by the transactivation domain) typically have at least some degree of fuzziness.

Adding to the difficulty of finding “druggable” sites to target, fuzzy features are challenging to capture with modern structural approaches like crystallography, as a static “snapshot” of the protein does not adequately reflect its structural multiplicity. NMR spectroscopy and molecular dynamics have shown significant promise in examining fuzzy complexes and their conformational ensembles (Schneider et al., 2015; Delaforge et al., 2018; Theisen et al., 2021), but critical information about any stable or metastable “cryptic” druggable pockets (Vajda et al., 2018; Mizukoshi et al., 2020) is often not a point of focus, even when they may be observable (Scholes and Weinzierl, 2016). The molecular recognition data that remains critical to structure-based drug design is not available for fuzzy TF complexes, stymying many drug discovery efforts (Scott et al., 2016). The logic and approaches of traditional drug discovery are thus currently ill-suited for targeting fuzzy TF PPIs, and a pivot to non-conventional approaches that take advantage of the “undruggable” properties of these interactions is necessary. In this Perspective, we briefly review approaches that have been taken to target these challenging interactions and discuss how they illuminate paths forward to take on fuzzy TF PPIs.

### The Direct Approach: Orthosteric Inhibition of Fuzzy TF PPIs

**FIGURE 2:**
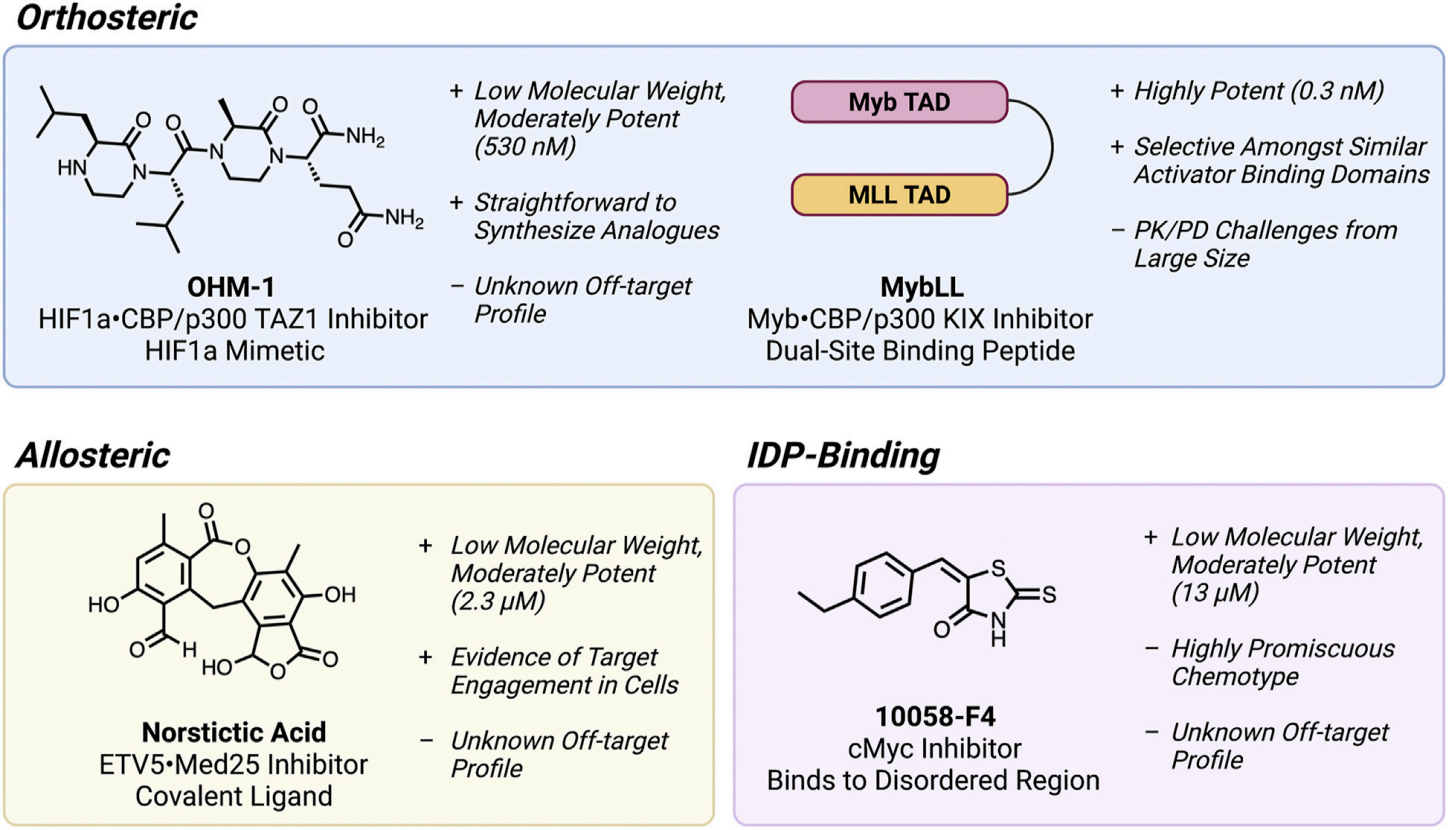
Examples of inhibitors of fuzzy TF PPIs and disordered TFs, annotated with key advantages, and disadvantages of each molecule. This figure was created using Biorender.com.

Orthosteric PPI inhibition, where a molecule directly blocks the PPI interface of one binding partner, is by far the most common approach attempted for targeting fuzzy TF PPIs. The discovery and development of these orthosteric inhibitors, on the other hand, has unfortunately proven to be an exceptionally challenging endeavor. Small molecule inhibitors have seen very limited success because of the large, flat interfaces they must disrupt (Figure 1B). Small molecule mimics of transactivation domains (TADs) have in some cases produced inhibitors of fuzzy TF•co-regulator PPIs, but potency and selectivity remain a serious issue overall (Best et al., 2004; Minter et al., 2004; Buhrlage et al., 2009). One outstanding example is the development of oligooxopiperazine mimics (Figure 2) of the HIF1α TAD, which reached sub-µM affinity for the TAZ1 domain of the coactivator CBP/p300 (Lao et al., 2014).

More often, relatively large molecules are required to span the extensive fuzzy PPI interfaces, which has been filled by peptide-based inhibitors. Inhibitors of the interactions of coactivator CBP/p300 with the TFs Myb and HIF1α interactions have been developed using inhibitory peptides based on the respective TF TADs (Henchey et al., 2010; Ramaswamy et al., 2018). Some of these, such as the Myb mimetic Mybmim have even seen significant *in cellulo* mechanistic characterization (Ramaswamy et al., 2018; Takao et al., 2021), but potency remains a major issue for therapeutic development because the parent TADs (and their respective mimics) generally have only moderate affinity (mid to low µM) for their targets. One recent example that overcame this challenge was the development of the CBP/p300 KIX inhibitor MybLL (Figure 2), which links the TADs of Myb, and MLL to form a sub-nM dual-site inhibitor of KIX (Joy et al., 2021).

Despite more success using peptide inhibitors, there are still major hurdles to advancing these molecules to the clinic. Peptide-based inhibitors of TF PPIs are generally quite large (several kDa) and rarely reach the nucleus without additional large cell-penetrating peptide motifs (Copolovici et al., 2014; Fu et al., 2014). Such large peptides are oftentimes difficult to advance to clinical studies due to several challenging pharmacokinetic and pharmacodynamic (PK/PD) properties, such as low stability and poor tissue distribution (Diao and Meibohm, 2013). Additionally, the most potent and selective peptide inhibitors are based on the relatively well-structured regions of the TAD when it is bound to the coactivator (Ramaswamy et al., 2018; Joy et al., 2021). Fuzzy TF interactions that have more limited structural formation are therefore not likely to benefit from this approach.

### Indirect Strategies: Allosteric Modulation by Conformational Capture

Given the limitations of the orthosteric inhibition strategies outlined above, the development of allosteric ligands has recently emerged as a promising approach to targeting fuzzy TF PPIs. Instead of directly targeting the TF•co-regulator surface, these approaches attempt to utilize possibly more druggable allosteric pockets on the co-regulator to control its conformational ensemble (Garlick and Mapp, 2020). Because structural flexibility is critical to the ability of these proteins to recognize diverse binding partners, limiting and/or redistributing the conformational ensemble with a ligand has the potential to not just completely inhibit TF•co-regulator binding, but also to subtly rewire the interactome to favor different binding partners.

A key model system where this approach has been investigated is the transcriptional coactivator Med25 and its TF binding partners. Compared to many of the TF•coactivator PPIs described previously, Med25 PPIs are considerably fuzzier, forming multiple highly distinct conformations at equilibrium in a manner unique to each TF (Henderson et al., 2018; Henley et al., 2020). A covalent allosteric ligand was first discovered by disulfide tethering, and was shown to enhance binding at one of the Med25 binding sites by modulating a dynamic allosteric loop (Henderson et al., 2018). However, disulfides are not stable in cells and this approach was limited to *in vitro* proof-of-concept. Recently, the natural product norstictic acid was shown to bind to a different dynamic loop and serve as a strong allosteric inhibitor of oncogenic ETV5 signaling (Garlick et al., 2021). This approach therefore appears to hold promise for targeting otherwise undruggable fuzzy TF PPIs.

### Insights From IDP-Binding Molecules

One major drawback of many of the approaches described thus far is that they almost exclusively involve targeting the TF binding partner because the TF itself is typically mostly intrinsically disordered (Figure 1A) (Liu et al., 2006). Developing ligands that directly bind the TF therefore requires advances in targeting intrinsically disordered proteins (IDPs).

In the TF-targeting field, the most well-known IDP binder is the Myc inhibitor 10058-F4 (Yin et al., 2003; Wang et al., 2007; Hammoudeh et al., 2009). This molecule binds to the intrinsically disordered monomeric Myc bHLH domain and inhibits its heterodimerization with its partner MAX to form a functional DBD. Because the therapeutic potential of targeting Myc is thought to be extremely high, several biophysical and structural studies have been undertaken to understand how 10058-F4 recognizes Myc (Hammoudeh et al., 2009; Heller et al., 2017). These efforts have shown that the molecule binds in a highly disordered and entropy-driven manner, where the IDP retains its disorder and the molecule “dances” around a set of residues that effectively constitute its “binding site.” The emerging hypothesis from these and similar efforts is that there is a degree of sequence specificity that some small molecules can use to bind IDPs in a selective manner.

Despite these advances in understanding small molecule•IDP interactions, it is important to note that the 10058-F4 scaffold contains an ene-rhodanine chemotype, known to many medicinal chemists as a common characteristic of “nuisance” compounds in high-throughput screening (Baell and Holloway, 2010; Dahlin et al., 2021). Rhodanines in particular are notorious for being reactive and/or nonspecific binders that are recalcitrant to the scaffold optimization needed to produce an efficacious drug. It is thus extremely unlikely that this molecule just acts as a Myc inhibitor *in vivo*. Several of the most biophysically well-characterized IDP-binding compounds reported to date also have similar such structural red flags (Akoury et al., 2013; Heller et al., 2020).

This begs the question: how well does the binding of a nonspecific molecule to an IDP represent the *ideal* binding modes that would enable the development of a potent and selective IDP-binding molecule? It will likely be more informative to study molecules that have been demonstrated to show some form of selectivity for their target, as there may be key differences in binding that lead to selectivity (e.g., partial folding of the IDP around the ligand) that will be critical to obtain a therapeutic effect without inducing off-target toxicity. While most *noncovalent* IDP binders reported thus far have minimal such selectivity data, several recently discovered selective *covalent* IDP binders could serve as a useful starting point for this purpose (Spradlin et al., 2019; Boike et al., 2021; Luo et al., 2021). Furthermore, developing computational methods to estimate the druggability of IDPs by focusing on structural characterization of metastable pockets (Zhang et al., 2015)—which can be observed via molecular dynamics simulations—is also of considerable value to the drug discovery community and does not necessarily require focusing on a very short list of well-validated IDP binders.

### The Issue of Validation

Even though some of these strategies have led to moderate successes, to the best of our knowledge, there are not any fully validated potent, and selective chemical probes (Frye, 2010; Arrowsmith et al., 2015) for fuzzy TF PPIs that have been described to date. We note that the bar for this is rather high, meaning many promising molecules (including the ones described in previous sections) that *do* bind and inhibit fuzzy TF complexes do not meet the bar because they have limited *in vivo* validation. This standard extends past the molecule producing the desired effect (inhibition of the fuzzy PPI *in vitro* and/or in cells) to include whether any phenotypic effects are caused by on-target mechanisms, and/or if the molecule acts by other additional means. The latter is surprisingly common to see in the transcription field, as there are *many* ways to illicit an effect on transcription that are unrelated to the desired mechanism (Kaelin, 2017).

Clearly this is a major point to be addressed by additional chemical and mechanistic biology efforts that run parallel to ligand discovery. Indeed, there are many modern approaches for validating target engagement and mechanism in living cells, from the determination of direct binding interactions between a small molecule and its targets *via* photoaffinity labeling (Flaxman and Woo, 2018), to CRISPR-based drug sensitivity screening methods that identify proteins that are required for the molecule to achieve its functional effects (Jost and Weissman, 2018). The longstanding “gold standard” of target validation is the identification of a resistance mutation in the target that abrogates the binding and phenotypic effects of a molecule. Given the abundance and increasing ease of utilizing these target identification approaches, campaigns to discover and develop modulators of fuzzy TF PPIs molecules should place more emphasis on obtaining clear evidence that lead molecules work through the desired mechanism rather than any number of off-target mechanisms.

## Discussion

In this Perspective, we have highlighted the major challenges associated with developing small molecule modulators of fuzzy TF PPIs and some of the approaches that have been taken to address these obstacles. However, the dearth of TF PPI inhibitors strongly indicates that new strategies are needed. To stimulate the development of new approaches, we point to what we believe are the major impediments facing current efforts, and call attention to some approaches that hold promise for taking on this target class.

The single most difficult challenge of targeting fuzzy TF PPIs comes from lack of high-quality structural information. While fuzzy complexes by their nature have diverse structural ensembles, there are specific points that need to be considered for drugging these complexes. First, how should structural and biophysical data be presented to the medicinal chemist? They might not be filled with inspiration and ideas from, for example, seeing plots of the conformational flexibility of a disordered region dancing across the binding surface of its partner. However, observation and characterization of metastable pockets opening on one of the partner proteins could be extremely useful for designing a molecule to bind that pocket. Many important lessons and strategies can and should be gleaned from recently developed computational approaches for identifying and characterizing druggable cavities in IDPs (Zhang et al., 2015).

Structure is also a challenge in terms of correctly representing the cellular context where a drug will be acting. The most detailed biophysical and structural data for fuzzy complexes are almost always obtained from reductionist approaches: with recombinantly expressed proteins in buffer and at concentrations orders of magnitude above their cellular concentration. While the challenge of getting similarly detailed biophysical data inside of living cells is extremely steep, it would be useful to encourage the adoption of integrated approaches to test biological models generated from biophysical data. For example, one of us recently published a paper describing a series of fuzzy TF PPIs that showed significant conformational differences from small sequence changes in the TF (Henley et al., 2020). It would be illuminating to next test the importance of these biophysical observations by making these sequence changes in cells and observing, for example, changes in gene expression or other phenotypes associated with the TF. Such information is critical to understanding which *in vitro* observations are most relevant to the function of the TF *in vivo*.

Finally, it is often the case that in drug discovery highly focused screening approaches are used, such as screening for inhibitors of a PPI or designing mimics of one of the binding partners. For fuzzy TF PPIs, this puts a lot of pressure on the need to be “right” about the critical characteristics of the interaction, such as the structure of a partner or the importance of a specific interaction interface. Furthermore, much of the *in vitro* optimization strategies that are used to design more potent drug candidates are often more challenging to implement because of both the data collection and the tenuous relationship between *in vitro* and *in vivo* structure (or regulatory state). On the other hand, untargeted phenotypic approaches are mired in false positives from molecules that act indirectly and/or promiscuously.

We propose that the answer to this problem is to use integrative approaches that treat direct *in vitro* data (e.g., ITC, NMR, etc.), indirect functional data (transcriptional output, phenotypic responses to treatment), and “intermediate” chemical biology data (chemoproteomics, target identification) as equals in determining which molecules to pursue and how to optimize them. Mechanistically unbiased approaches for discovering molecules, such as high-throughput binding screens, can also be extremely useful because they alleviate the need to choose the “right” way to drug a given TF•co-regulator PPI. Instead, judicious choice of secondary assays can allow opportunities to present themselves based on the properties of screening hits. For example, while a recent discovered fragment molecule that binds directly to the “undruggable” beta-catenin does not inhibit its binding to the TF Tcf4 (Kessler et al., 2021), it appears to be well positioned for development into a bifunctional proteolysis targeting chimaera (PROTAC) to instead inhibit Tcf4 function by degrading beta-catenin.

Transcription factors and their fuzzy protein-protein complexes hold enormous potential as drug discovery targets across a wide range of diseases. While several promising approaches for targeting fuzzy TF PPIs have emerged, the field is still a way off from producing well-validated chemical probes and drugs. The expertise of the biophysical-leaning fuzzy PPI and IDP community melded with modern chemical biology approaches could make a potent recipe for taking this problem head on.

## Data Availability

The original contributions presented in the study are included in the article/Supplementary Material, further inquiries can be directed to the corresponding author.
